# Serum Fatty Acids, Traditional Risk Factors, and Comorbidity as Related to Myocardial Injury in an Elderly Population with Acute Myocardial Infarction

**DOI:** 10.1155/2016/4945720

**Published:** 2016-02-18

**Authors:** Kristian Laake, Ingebjørg Seljeflot, Erik B. Schmidt, Peder Myhre, Arnljot Tveit, Harald Arnesen, Svein Solheim

**Affiliations:** ^1^Center for Clinical Heart Research, Department of Cardiology, Oslo University Hospital, Ullevål, 0450 Oslo, Norway; ^2^Faculty of Medicine, University of Oslo, 0316 Oslo, Norway; ^3^Center for Heart Failure Research, University of Oslo, 0316 Oslo, Norway; ^4^Department of Cardiology, Aalborg University Hospital, 9000 Aalborg, Denmark; ^5^Department of Cardiology, Akershus University Hospital HF, 1478 Lørenskog, Norway; ^6^Department of Medical Research, Vestre Viken Hospital Trust, Bærum Hospital, 1346 Rud, Norway

## Abstract

*Background.* Epidemiological and randomized clinical trials indicate that marine polyunsaturated n-3 fatty acids (n-3 PUFAs) may have cardioprotective effects.* Aim.* Evaluate the associations between serum fatty acid profile, traditional risk factors, the presence of cardiovascular diseases (CVD), and peak Troponin T (TnT) levels in elderly patients with an acute myocardial infarction (AMI).* Materials and Methods.* Patients (*n* = 299) consecutively included in the ongoing Omega-3 fatty acids in elderly patients with myocardial infarction (OMEMI) trial were investigated. Peak TnT was registered during the hospital stay. Serum fatty acid analysis was performed 2–8 weeks later.* Results.* No significant correlations between peak TnT levels and any of the n-3 PUFAs were observed. However, patients with a history of atrial fibrillation had significantly lower docosahexaenoic acid levels than patients without. Significantly lower peak TnT levels were observed in patients with a history of hyperlipidemia, angina, MI, atrial fibrillation, intermittent claudication, and previous revascularization (all *p* < 0.02).* Conclusions.* In an elderly population with AMI, no association between individual serum fatty acids and estimated myocardial infarct size could be demonstrated. However, a history of hyperlipidemia and the presence of CVD were associated with lower peak TnT levels, possibly because of treatment with cardioprotective medications.

## 1. Background

Coronary heart disease (CHD) including myocardial infarction (MI) is one of the leading causes of mortality in the Western world and the incidence increases with advancing age [1]. The cardioprotective effects of marine polyunsaturated n-3 fatty acids (n-3 PUFA) have been extensively studied, and earlier epidemiological [2] and large scale clinical trials [3, 4] have shown beneficial effects, although these are not without controversy [5]. In the landmark GISSI-Prevenzione-trial [3], secondary prevention with 1 g/day of n-3 PUFA supplementation resulted in a 45% decrease in sudden death, but with no effect regarding nonfatal MI. Later trials have contradicted these results [6, 7] showing no effect of n-3 PUFA on clinical endpoints; however they have been criticized for lacking statistical power [8] and for using inadequate treatment dosages [9]. The combined treatment with both statins and n-3 PUFA after MI has shown improved cardiovascular (CV) outcomes compared to treatment with statins alone [10], and treatment with n-3 PUFA within 14 days of a first MI has been associated with a 32% risk reduction of all-cause mortality in patients on concurrent lipid-lowering, antihypertensive, and antiplatelet treatment [11]. N3-PUFA supplementation has also shown improved outcome in heart failure [12, 13].

Mozaffarian et al. examined a large cohort of elderly subjects without prevalent CHD and found that higher levels of very long chain marine n-3 PUFAs in plasma were associated with a lower total mortality and especially fewer cardiac deaths [14]. There are, however, few published studies on circulating fatty acids concentrations and their association to CV events, and some studies are based on diet-questionnaires for estimation of intakes of marine n-3 PUFA [15, 16]. Infarct size is important for mortality and morbidity, and while there are no data in humans regarding the effect of n-3 PUFA on infarct size, one study has shown that rats fed with dietary fish oil for 8 weeks had a significantly reduced infarct size [17].

Studies on mechanisms have revealed n-3 PUFAs triglyceride lowering and anti-inflammatory and antiarrhythmic and platelet inhibiting and blood pressure lowering effects, including improved endothelial function and plaque stability [18]. The Troponin T cardiac biomarker (TnT) has been shown to correlate with infarct size [19] and peak TnT levels have been shown to be strongly correlated with infarct size assessed by single-photon emission computed tomography [20].

The aim of our study was to evaluate the serum fatty acid profile, traditional risk factors, and relevant cardiovascular disease states as related to myocardial injury estimated by peak TnT in an elderly population of patients that have experienced an acute MI. Our hypothesis was that the pattern of serum fatty acids as well as traditional risk factors and previous cardiovascular disease states would influence peak TnT levels. To our knowledge this has not been previously examined.

## 2. Materials and Methods

### 2.1. Study Design

The present study is a substudy of the OMEMI trial with a design that has previously been described in detail [21]. In short, the study is a prospective randomized placebo controlled multicenter trial evaluating the effect of a 2-year intervention with n-3 PUFA supplementation (1.8 g/d) on cardiovascular endpoints in elderly patients, age 70–82 years, that have suffered an acute MI (type 1–4), and without comorbidity thought to be incompatible with study drugs or a 2 year follow-up. Compliance is evaluated by serum levels of fatty acids recorded at baseline and at 24 months.

Hypertension (HT) was defined as previously known systolic blood pressure >140 and/or diastolic blood pressure >90 mmHg or treatment for HT. Diabetes was defined as being insulin dependent or noninsulin dependent. Hyperlipidemia was defined as being previously treated for/or diagnosed with dyslipidemia. Atrial fibrillation (AF) was defined as a history of ECG-documented paroxystic, persistent, or permanent AF and smokers were defined as current smokers. The study was carried out in compliance with the Helsinki Declaration and approved by the Regional Ethics Committee. All subjects gave their written informed consent to participate (ClinicalTrials.gov, NCT01841944).

The present results are based on participants included from November 2012 to October 2014.

### 2.2. Laboratory Methods

Peak TnT levels were registered during the index MI. Further data collection and serum fatty acid analyses were performed in blood samples obtained 2–8 weeks later at inclusion in the study. Blood samples were obtained in fasting condition (>10 h) by standard venipuncture between 08:00 and 11:00 am, before daily intake of medication. Serum was prepared by centrifugation within one hour at 2500 g for 10 min and kept frozen (−80°C) until analyzed. Routine blood samples were determined with conventional methods. Electrochemiluminescence technology for quantitative measurement was used for repeated measures of TnT (3rd-generation cTroponinT, Elecsys 2010, Roche, Mannheim, Germany) with interassay coefficient of variation (CV) of 7%. NT-proBNP was measured in serum using Elecsys proBNP sandwich immunoassay on Elecsys 2010 (Roche Diagnostics, Indianapolis, USA) with CV of 7%.

Serum phospholipids fatty acid composition was analyzed by gas chromatography at the Lipid Research Laboratory, Aalborg University Hospital, Denmark. Briefly, serum lipids were extracted by the Folch procedure [22]. Serum 500 *μ*L was mixed with 5 mL chloroform-methanol 2 : 1 containing 50 *μ*L/mL butylated hydroxytoluene as antioxidants and shaken. After addition of 750 *μ*L 0.9% sodium chloride, the tubes were mixed and centrifuged at 3220 g for 10 minutes. The lower organic phase containing total lipids was collected and a second extraction was performed on the remaining protein disc. The organic phase was then dried under nitrogen for 45 min at 40°C and dissolved in 1 mL chloroform. Separation of phospholipids fatty acid fraction from total lipids was performed by the method of Burdge et al. [23], with the modification that all types of phospholipids were sampled (see also below). The tube containing the organic phase was transferred to Agilent Bond Elut NH2 column 200 mg (Agilent Technologies, US) preconditioned with 4 mL hexane. The phospholipids fatty acid fraction was eluted with 2 mL chloroform-methanol 3 : 2 followed by 2 mL of methanol and then dried under nitrogen for 1 hour at 40°C. Methylation of the fatty acids was performed before being analyzed by gas chromatography using Varian 3900 gas chromatograph equipped with a CP-8400 autosampler, a flame ionization detector, and a high-polarity polyester CP-Sil 88 60 m ×0.25 mm capillary column (Varian, Middleburg, Netherlands).

The serum content of linoleic acid (LA) (18:2, n-6), arachidonic acid (AA) (20:4, n-6), eicosapentaenoic acid (EPA) (20:5, n-3), and docosahexaenoic acid (DHA) (22:6 n-3) was expressed as a percent of total fatty acids (wt%) and the CVs were 0.4%, 0.6%, 1.1%, and 1.8%, respectively.

### 2.3. Statistics

As most data had a skewed distribution, the results are presented as median values (25 and 75 percentiles) unless otherwise stated. Nonparametric statistics were used throughout. For group comparison, Mann-Whitney *U* test was used for continuous variables. Analyses of correlations were performed with Spearman rho. A two-tailed value of *p* ≤ 0.05 was considered statistically significant. The statistical analyses were performed with IBM SPSS Statistics, version 21.0.0.2 (IBM, New York, USA).

## 3. Results

**Figure 1:**
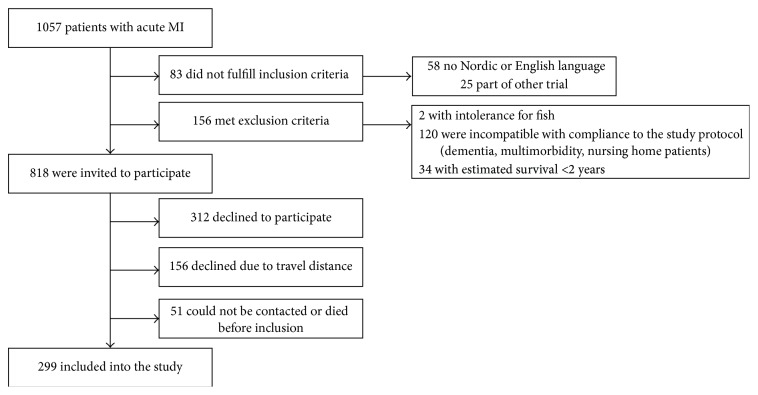
Flowchart of patients.

Flowchart for the inclusion of patients in the present cohort is shown in Figure 1. Characteristics of the population at inclusion are shown in Table 1. Median age was 75 years, 74% were male, 61% had hypertension, 48% had hyperlipidemia, 30% had a previous myocardial infarction, 23% had diabetes, 14% were current smokers, and 13% had atrial fibrillation.

### 3.1. Serum Fatty Acids in relation to Myocardial Injury

We focused primarily on the levels of AA and LA n-6 and marine EPA and DHA n-3 fatty acids, as well as the AA + LA/EPA + DHA (n-6/n-3) ratio, as these are the most relevant for CHD. Median values are shown in Table 2.

We found no significant correlations between peak TnT levels and any of the fatty acids or the n-6/n-3 ratio (Table 3). We also evaluated any difference in peak TnT levels between the lowest (10th) and highest (90th percentile) of serums EPA + DHA and AA + LA and n-6/n-3 ratio, but the observed results were insignificant (all *p* > 0.7). There was also no correlation between any of the fatty acids or the n-6/n-3 ratio and NT-proBNP, an indicator of heart failure.

Significantly higher serum levels of EPA and DHA in participants who reported previous intake of n-3 PUFA supplements (*n* = 135) compared to those who did not (*n* = 161) were observed, resulting in a significant difference in the n-6/n-3 ratio (3.0 versus 4.1, *p* < 0.001). However, no difference in peak TnT levels between these two groups (644 versus 711 ng/L, *p* = 0.908) was revealed. Furthermore, there were no significant differences in levels of different fatty acids between patients with ST-segment elevation myocardial infarction and non-ST-segment elevation myocardial infarction.

In patients with known atrial fibrillation (*n* = 38), significantly lower DHA levels (5.1 versus 5.7 wt%, *p* = 0.005) and a higher n-6/n-3 ratio (4.2 versus 3.5, *p* = 0.028) were observed.

There were no significant differences in any of the n-6 or n-3 fatty acid levels between genders or in patients with or without a previous MI or smokers and nonsmokers. Lower levels of AA were found in diabetics versus nondiabetics (10.7 versus 9.2, *p* < 0.001).

### 3.2. Peak TnT Levels in relation to Traditional Risk Factors and Cardiovascular Disease States

Comparisons between peak TnT (ng/L) levels during index hospitalization and CVD risk factors and relevant disease entities are shown in Table 4.

Significantly lower TnT levels were observed in patients with a history of hyperlipidemia (*p* = 0.018), angina pectoris (*p* < 0.001), previous MI (*p* = 0.003), atrial fibrillation (*p* = 0.007), and claudication (*p* = 0.015) and also for participants that have previously undergone revascularization procedures (*p* = 0.003). However, no significant differences were observed between genders or in patients with or without hypertension or diabetes or smokers versus nonsmokers. We found no correlations between infarct size estimated by peak TnT and age or body mass index (BMI). However, a strong positive correlation between infarct size and NT-proBNP (*r* = 0.57, *p* < 0.001) measured at inclusion was found.

With regard to previous CVD states and concurrent medical treatment (data on medication use available in 134), we found that, for previous angina, 77% were on aspirin, 67% were on statins, and 72% on were beta blockers; for previous MI, 76% were on aspirin, 79% were on statins, and 79% were on beta blockers; and, for previous AF, 59% were treated with oral anticoagulants.

## 4. Discussion

The main finding in the present study was that no significant association could be demonstrated between estimated infarct size by peak TnT and the relative content of long chained marine n-3 PUFAs in serum phospholipids in elderly subjects with an acute MI. There was, however, a significant inverse correlation between serum levels of DHA and the history of atrial fibrillation, and patients with a history of hyperlipidemia and relevant CVD entities presented with significantly lower peak TnT levels. Finally, a strong correlation was observed between peak TnT during the index infarction and NT-proBNP at inclusion.

Long chain marine n-3 PUFAs have several different effects that together are considered cardioprotective [18, 24]. There is limited data on their ability to modulate infarct size and protect ischemic myocardium during an acute MI. Even though previous animal studies may suggest that n-3 PUFAs have the ability to reduce infarct size, it could not be demonstrated in our study. It has been shown that cardiomyocytes generate significant amounts of reactive oxygen species (ROS) during ischemia, which may contribute significantly to cellular injury [25]. Therefore, the possibility for n3-PUFAs to provide acute cardiovascular protection and reduce the extent of myocardial injury is suggested to be through their anti-inflammatory and antioxidant functions [26–28].

An earlier study revealed that EPA significantly reduced infarct size in rabbit hearts, mainly dependent on its ability to increase nitric oxide (NO) production and opening of calcium-activated potassium channels [29]. It has also been shown that ischemia-reperfusion injury was reduced in rats after 8 weeks of n-3 supplementation [30]. A study administering DHA acutely before induction of coronary ischemia in Sprague-Dawley rats has revealed similar results, with lowering of lipid peroxides and a reduction in myocardial infarct size and creatine kinase release [31]. In a recent study it has also been demonstrated that Resolvin D1, a DHA metabolite, confers myocardial protection and reduces infarct size via mechanisms involving the PI3K/Akt signaling pathway and attenuation of caspase-3 and caspase-8 activation [32]. In two human trials, cardiac patients pretreated with a high-dose of n-3 PUFAs before planned invasive treatment did display lower serum levels of cardiac biomarkers following percutaneous coronary intervention (PCI) [33] and coronary artery bypass surgery [34] compared to controls. Our population had an intake of dietary marine n-3 PUFAs prior to enrolment of a median n-6/n-3 ratio of 3.6, which is considered to be very low. It has been noted that background n-3 fatty acid intake could change the cardiovascular outcomes in trials with different populations receiving the same treatment doses [35] and that a different n-6/n-3 ratio would influence infarct size [36]. Although we found no significant difference in peak TnT levels between those with lowest (10th) versus highest (90th) percentiles of n-6 and/or n-3 fatty acids, it might be suggested that the results would be different in a population with a higher n-6/n-3 ratio. Almost half of our study population was on aspirin and statin treatment; thus any effect of n3-PUFAs on infarct size may have been masked.

Interestingly, significantly lower levels of DHA in patients with a history of atrial fibrillation were found. In the landmark GISSI-Prevenzione-trial, it was shown that n-3 fatty acid supplementation led to a reduction in sudden cardiac death, pointing to n-3 PUFAs antiarrhythmic potential [3, 37]. Our findings are in accordance with Wu et al. who found that higher circulating levels of DHA in older adults were associated with lower risk of incident AF [38]. Furthermore, in a large, albeit younger, Danish cohort, data showed an inverse, however, not statistically significant, association between the development of AF and n-3 PUFAs in adipose tissue [39]. In this cohort a U-shaped association was established between incident AF and marine n-3 fatty acid consumption, especially for EPA and DHA, with lowest risk close to the median intake [40]. McLennan did reveal that DHA is increasingly accumulated in the myocardial cell membrane over EPA, which might point to DHA as the most potent fatty acid regarding the antiarrhythmic effects of n-3 PUFAs [41]. Still, its role in AF seems unclear. A meta-analysis evaluating 16 studies did not find any benefit of n-3 PUFA supplementation on secondary prevention or incidence of new AF after cardiovascular surgery [42], and a recent trial randomizing patients with previous known atrial fibrillation to 4 g/day n-3 PUFA or control did not show any reduction in AF recurrence [43].

We also found significantly lower TnT levels in those with a history of hyperlipidemia, especially for angina and other relevant CVD states. Kloner et al. did show that patients experiencing an AMI with a history of angina were more likely to have smaller creatine kinase-determined infarcts compared to those without [44]. The authors consider this to be related to the effect of ischemic preconditioning, which could explain our findings. It should also be noted that patients with previous diseases like hyperlipidemia, MI, and intermittent claudication in our study are likely to use both platelet inhibitors and statins, which could affect the size and extent of myocardial infarction. A retrospective study has shown that recent aspirin use was associated with smaller myocardial infarct size [45] and similar effects have been reported after clinical trials with beta blockers [46]. Furthermore, pretreatment with statins (rosuvastatin) prior to PCI is shown to reduce periprocedural myocardial injury in patients with ACS and ST-segment elevation myocardial infarction [47, 48]. Data on medication use prior to index infarction was available in almost half of our population, and we found that patients with previous CVD were well treated with the majority on aspirin, statin, and beta blocker. We could therefore speculate that previous drug treatment may have limited the infarct size in our participants with risk factors for CVD and relevant disease states.

Finally, the rather strong correlation between peak TnT during the index infarction and NT-proBNP at inclusion 2–8 weeks later is to be expected as TnT as a marker of infarct size should be expected to associate with NT-proBNP as a predictor of myocardial injury and long-term prognosis [49].

## 5. Strengths and Limitations

The strengths of the study are attributed to the rather large population and the serum phospholipids analysis of n-3 fatty acids which is considered an important addition to dietary questionnaires. It could be argued that our measure of serum phospholipids, 2–8 weeks after index hospitalization, would not provide a correct reflection of the situation during the AMI. Although we did find significantly higher levels in patients previously reporting intake of n-3 PUFA supplements versus those who did not, confirming our belief that elderly patients would not largely change their dietary habits in a relatively short time, therefore our results should be considered representative. Our population also has a previously high intake of marine n-3 fatty acids. Also, we determined the relative fatty acid composition of phospholipids in serum and not absolute concentrations of individual fatty acids. Lastly, although peak TnT has shown strong correlation with infarct size assessed by radionuclide imaging, it is not considered the gold standard for this estimation. Also, peak TnT levels may not be the absolute peak as the blood samples have been taken according to clinical guidelines during the MI, and not through a standardized method in the protocol.

## 6. Conclusions

In an elderly population with AMI, no significant association between serum fatty acids and estimated infarct size could be demonstrated. However, an inverse correlation was noted between serum levels of DHA and a history of atrial fibrillation. Hyperlipidemia and the presence of CVD were associated with lower peak TnT levels, probably because of treatment with cardioprotective medication at the time of the AMI.

## Figures and Tables

**Table 1:** Baseline characteristics of the study cohort. Data presented as percentages or median values (25 and 75 percentiles).

Age (y)	75 (72, 78)
Gender (male/female) (%)	73.9/26.1
Smoker (current/previous) (%)	13.7/46.8
Body mass index (kg/m^2^)	25.6 (23.8, 28.3)
STEMI (%)	31.4
3-vessel disease (%)	21.3
Peak Troponin T level (ng/L) @ index MI	700 (153, 2500)
NT-proBNP (*n* = 173)	75.0 (33.0, 162.5)
History of hypertension (%)	182 (60.9)
History of hyperlipidemia (%)	143 (47.8)
History of atrial fibrillation (%)	38 (12.7)
Previous myocardial infarction (%)	90 (30.1)
Previous heart failure (%)	16 (5.4)
Previous diabetes (%)	69 (23.1)
Medication @ index MI (*n* = 134):	
Aspirin (%)	64 (47.8)
Other platelet inhibitors (%)	7 (5.2)
Anticoagulation (%)	19 (14.1)
Beta blocker (%)	61 (45.5)
ACE-I/AT II blocker (%)	61 (45.5)
Calcium channel blocker (%)	30 (22.4)
Statin (%)	68 (50.7)
Diuretic (%)	38 (28.4)
Nitrates (%)	8 (6.0)
n-3 PUFA supplements (%) (*n* = 296)	135 (45.6)

ACE-I/AT II: angiotensin-converting enzyme inhibitors/angiotensin II receptor blockers; STEMI: ST-segment elevation myocardial infarction.

**Table 2:** Median values of selected n-3 fatty acids (% of total fatty acids in serum phospholipids) at baseline.

Eicosapentaenoic acid (EPA) n-3 (20:5) (wt%)	2.4
Docosahexaenoic acid (DHA) n-3 (22:6) (wt%)	5.6
Linoleic acid (LA) n-6 (18:2) (wt%)	19.0
Arachidonic acid (AA) n-6 (20:4) (wt%)	9.6
n6/n3 ratio	3.6

**Table 3:** Coefficients of correlations^1^ between the selected fatty acids (% of total fatty acids in serum phospholipids) and peak Troponin T (ng/L) during index myocardial infarction.

Eicosapentaenoic acid (EPA)	*r* = −.052
n-3 (20:5)	*p* = 0.369
Docosahexaenoic acid (DHA)	*r* = .060
n-3 (22:6)	*p* = 0.303
Linoleic acid (LA)	*r* = .021
n-6 (18:2)	*p* = 0.717
Arachidonic acid (AA)	*r* = .014
n-6 (20:4)	*p* = 0.808
n6/n3 ratio	*r* = .002
	*p* = 0.966

^1^Spearman's rho are given.

**Table 4:** Comparisons of peak Troponin T (ng/L) levels and the presence of cardiovascular risk factors and relevant disease entities.

	−	+	*p*
Previous diabetes (*n* = 69)	732.5	664.0	0.892
History of hyperlipidemia (*n* = 143)	916.5	496.0	**0.018**
History of hypertension (*n* = 182)	1009.0	584.0	0.057
Previous angina (*n* = 88)	923.0	251.0	**0.001**
Previous claudication (*n* = 27)	795.0	237.0	**0.015**
Previous myocardial infarction (*n* = 90)	910.0	344.0	**0.003**
Previous revascularization (*n* = 83)	874.5	297.0	**0.003**
History of atrial fibrillation (*n* = 38)	778.0	153.0	**0.007**
Current smoker (*n* = 41)	767.5	519.0	0.292
Previous n-3 PUFA supplementation (*n* = 135)	711.0	644.0	0.908

+ denotes presence of risk factors or disease entities.

− denotes absence of risk factors or disease entities.
